# Creative patient and public engagement to amplify the voices of people living with eczema

**DOI:** 10.12688/wellcomeopenres.26101.1

**Published:** 2026-09-26

**Authors:** Alison Mayne, Keira Tucker, Catherine Street, Sarah Rose Graber, James Brook, Liam Russell, Dee Davison, Claire Doyle, Eczema Outreach Support Youth Panel, Beverley Hood, Sara Brown

**Affiliations:** 1 Edinburgh College of Art, The University of Edinburgh College of Arts Humanities and Social Science, Edinburgh, Scotland, EH3 9DF, UK; 2 Summerhall, ASCUS Art & Science, Edinburgh, EH9 1PL, UK; 3 James Brook Design, Edinburgh, UK; 4 Stomping Ground Creative, Dumfries, Scotland, UK; 5 Institute of Genetics and Cancer, The University of Edinburgh College of Medicine and Veterinary Medicine, Edinburgh, Scotland, EH4 2XU, UK; 6 Eczema Outreach Support, Linlithgow, Scotland, EH49 7EZ, UK; 7 Department of Dermatology, NHS Lothian, Edinburgh, Scotland, EH3 9HX, UK

**Keywords:** art; atopic eczema; creative; multidisciplinary; public-patient participation

## Abstract

**Background:**

Qualitative research has captured human experiences of suffering associated with atopic eczema. We aimed to convey this information beyond academic manuscripts to a broader audience, using ambitious, multidisciplinary patient and public engagement, led by creative researchers.

**Methods:**

We worked with patient communities to find ways to amplify eczema voices, using previously reported and newly collected quotes describing their experiences. The resulting “Surface Echoes” project combined audio and visual methods, developed by a collaborative team of creatives, working with stakeholder participants.

**Results:**

A script for reading aloud in small groups, and a digital audio recording were created through a process of collaging the collected quotes, curated by the patient communities, creatives and researchers. Themes presented in this work include visual appearance and mental burden of embarrassment or shame (“ *it’s a kind of social handicap…*”); pain and itch; lack of clarity around treatment (“ *I think this ‘trial and error’ is a very primitive form of medicine*”); challenges of communicating with healthcare professionals; and the morbidity associated with sleep deprivation (“ *It’s impossible to be on sick leave just because you are tired. So I carried on throughout the Spring and then I completely collapsed*”). The curated quotes also report positive healthcare experiences, indicative of good practice: “ *I felt listened to … it was a conversation*”. Project participants used creative research methods to develop imaginaries of better futures, for example, “ *… sunshine soup”* and *“… consistent advice*”. The materials have been used with stakeholders including medical and nursing professionals, industrial and academic researchers, and the public.

**Conclusions:**

This work has been designed to raise awareness, promote discussion, reduce stigma and increase understanding of eczema. The presentation to stakeholder groups prioritised by the participants whose voices are amplified was designed to improve empathic discussion with professionals; it may also inform future research.

## Plain Language summary

The “Surface Echoes” project produced a script and audio to amplify views and experiences of people living with the itchy, painful skin condition eczema. The work was conducted by a team based in Edinburgh, Scotland, including patients, members of the public, scientists and healthcare workers, led by creative researchers. Themes included participants’ negative experiences of embarrassment and shame, challenging communications, uncertainty about treatments, physical pain, and exhaustion from sleep deprivation. Positive experiences were also reported, and participants shared imaginations of a better future. The script and audio are available for dissemination to stakeholders, including healthcare professionals and researchers.

## Introduction

Atopic eczema (synonymous with atopic dermatitis) is an itchy inflammatory skin condition affecting approximately 20% of school-age children in the UK and up to 10% of adults ^1^; there are an estimated 129 million individuals with atopic eczema worldwide. ^2^ Moderate-severe eczema has a profound impact on personal and social life, including chronic sleep deprivation because of pruritus and skin trauma through scratching. A young child may struggle to concentrate at school and an adult experiences fatigue, as well as social stigma. ^3^ There is an increased prevalence of neurodevelopmental disorders such as ADHD, as well as anxiety and depression in children and adults. ^1^ Atopic eczema ranks fifteenth among nonfatal diseases measured by disability-adjusted life-years and it has the highest disease burden among skin diseases. ^4^ Eczema is responsible for more disability-adjusted life years than other severe inflammatory disorders, such as arthritis. ^5^ However, there remains a considerable degree of misunderstanding about this very visible skin condition and patients report concerns that healthcare professionals do not always have a good understanding of the condition, or empathetic approach. ^6^

Qualitative research has effectively recorded the range of experiences of people living with eczema. ^7^ But this research, which includes some powerful and moving quotes describing personal challenges in everyday life, is captured within academic, research publications in a format that is not readily accessible to the general public. Our work aimed to share existing quotes, whilst also collecting and amplifying new voices of people affected by eczema, to allow their expression in a form that can be anonymous and/or public, through ambitious, multidisciplinary patient and public engagement led by creative researchers.

## Methods

### Multidisciplinary team

We formed a multidisciplinary team with a diversity of skills and perspectives to work in this challenging and sensitive area of human experience and expression. The team composition and contributions are summarised in Table 1.

**Table 1.** Multidisciplinary team composition. Team members (initials represent the respective author) and their contributions to the “Surface Echoes” project.

Team member	Contribution
Practice-based creative researcher (BH)	Design and collaborative creation of “Surface Echoes” resource
ASCUS Art & Science organisation (KT)	Project management, communications, documentation, workshop development and facilitation
Creative professionals (CS, JB, CD, SRG, LR)	Artists, performers, designer, and sound designer, for collaborative creation of “Surface Echoes” resource
Patient and public involvement and engagement (DD)	Expertise in methods and best practice of PPIE
Eczema Outreach Support Youth Panel	Eczema patient involvement in creation of “Surface Echoes” resources
Public volunteers with lived experience of eczema	Participation in creative workshops inputting to creation and review of “Surface Echoes” resources
Clinical academic dermatologist (SB)	Oversight of clinical and scientific research aspects of the final resource
Administrative support (AM)	Meeting organisation, ethics application, workshop facilitation

### Curation of quotations describing the lived experience of eczema

Previously published quotations were collated from qualitative studies and academic review articles, ^7– 22^ describing the complexity of real-life experiences of chronic eczema. These described the far-reaching impact beyond a typical preconception of eczema as only an itchy skin condition. We recognised that bringing this material together created a significant archive of qualitative material, lived experiences of the condition eczema, drawn from many years of research across a variety of communities that included perspectives from patients, carers, and health professionals.

To select the most relevant quotations and enrich the resource with current experience, we created a series of participatory research workshops that brought the original quotation materials to different stakeholder groups. Two cohorts participated in workshops: seven adults with lived experience of eczema recruited through the University email and our project partners; and the Eczema Outreach Support Youth Panel, comprising six individuals aged 16–25 years. The individuals contributing to the workshops had current or previous experience of eczema with a wide range of severity.

### Participatory creative research workshops

The participatory creative workshops were designed and facilitated by team members with expertise in public and patient participatory involvement and engagement (AM, DD, KT, BH, CS and CD). A variety of creative participatory methods ( Table 2) were employed to open-up discussion about lived experience, using content in a more poetic way. Previously published qualitative material was considered and new experiential content was developed and recorded, using the central guiding principle of mutual respect to value and not diminish any lived experience.

**Table 2.** Creative research methods used in participatory workshops. Methods and description of their use in the “surface echoes” workshops.

Method	Description
Chatterboxes	Printed paper was used to create chatterbox ^23^ toys to allow participants to explore questions and possible answers to prompt further discussion
Quotation cards discussed and placed within proximity circles	An A1 piece of paper was printed with concentric circles radiating out from a centre point labelled ‘closest to my experience’ towards an outer circle labelled ‘furthest away from my experience’. Participants used the circles to guide placement of selected quotations printed on cards.
Storytelling	Workshop participants were encouraged to write down their own stories, using words or sketched images according to their preference.
Material exploration	Participants in an online workshop were requested to leave their screens and search their home environments, to select a material item which in some way reflected some aspect of their feeling or experience of eczema. Materials were brought back to show to the group online, sharing an explanation of why the item had been selected.
Word cloud generation	The free online tool Mentimeter ( https://www.mentimeter.com/) was used to generate word clouds from participants’ responses, to share experiences and promote discussion.
AI text-to-speech	AI generated speech was used to try out extracts of stories from the workshop participants, and whether the AI voices added or took anything away from the meaning through their digital diction.
Future island imaginaries	Workshop participants were provided with an A3 paper outline of an island and a range of materials with which to map social and geographical details onto the island, as a tool to imagine and describe their perfect future life without eczema.
Laboratory workshop	Viewing of individuals’ skin cells under the microscope, to give a unique new perspective.

Material exploration was used as a creative exercise to stimulate conversations about the experience of life with a skin condition. In an online workshop, participants showed and talked about materials that they had selected in identification with their skin, or representing an aspect of their experience of eczema.

Lived experience of eczema brings a substantial burden, so we employed an exercise in imagination to engage thinking in a positive future-focused way. We asked the stakeholder groups to consider a ‘future island’, in which the ideal concept is life without eczema: what could happen in the midst of that journey, from where we are now to having eczema gone from life? This was inspired in part by the involvement of a team member (SB) whose expertise is in molecular genetic research for therapy development aimed to prevent and treat atopic eczema for a better future.

Each of the activities listed in Table 2 were recorded and transcribed as further textual material, creating an evolving multifaceted archive of lived experience of eczema.

### Collaborative creation of text and quotation collage to form a script

Text collaging was used as a method to address the challenge of curating and editing such a large volume of textural material from the different sources and to bring together original quotes from research papers, material generated from working with groups to evaluate their own responses, including more poetic versions of experience and the future-facing imaginaries. Two artists worked in close collaboration (CS and BH) to reconcile the substantial corpus of material. The artists employed a meditative process, drawing together associations between images and text, to work through the materials and make connections. This gentle collaborative creative process involved close reading aloud, in turns, at a slow, attentive pace. Close reading was used to select text based on emotional and poetic resonance and connection to the overall concepts of the project. Selected text underwent physical editing in which words and phrases were literally cut out, and the resulting paper fragments were tacked down onto a long roll of paper. The word cuttings were organised into themes, for example ‘sleep’, ‘care’, ‘creams’. This was necessarily an unwieldy process, in which the working draft of the text collage reached 12 m in length, before being edited and re-edited to reduce the length in an iterative process of reading and re-reading together, negotiating every line. This created a long, slow and deeply considered process in which the artists pieced together, line by line, what was to become a script. The quotes were ultimately formed into a script to be read aloud, in recognition that the workshop activities of people reading, recounting and discussing the quotes was a meaningful process in itself. This resulted in the final work to be made in a form to be read aloud in a group setting, to recreate the powerful experience of workshop participants sharing their insights.

Importantly, the editing process was not restricted to input by creative artists. Extracts of the collage material were taken to the Eczema Outreach Support Youth Panel in a follow-on participatory workshop, supported by a theatre educator (CD). This stakeholder group reviewed and commented on the narrative, tone, and shape of the overall material, giving advice on methods of reading and performance. Finally, another professional theatre practitioner (SRG) contributed a dramaturgical role, to steer the overall form and narrative of the full script through a process of three artists (CS, BH and SRG) closely reading out in turns, whilst physically editing the text collage into a script.

### Testing of material with researchers and other stakeholder groups

The resulting material was taken to stakeholder groups composed of healthcare professionals, scientists, arts and health researchers to test its use as a collective reading experience. This group testing allowed an exploration of form, as well as an assessment of audiences’ engagement with the work, to refine our future engagement for dissemination.

### Creation of visual and audio versions of script to maximise accessibility

Graphic design expertise (JB) created A4 versions of the script for use in group-reading activity. The original script was visually tweaked and adapted into a printable PDF, intended to be read aloud together as a collective experience of reading and listening to other people’s voices, while keeping the aesthetic of the original collage. A complementary audio version of the script was recorded, edited and composed by a sound designer (LR), featuring five professional voice actors of different ages and genders and with regional accents from across the UK. This diversity was selected to give resonance across a range of communities and to portray the diversity of people affected by eczema. Finally, the original text collage was framed for public display.

## Results

### Participatory workshops

A selection of photos illustrates aspects of the creative processes used in our participatory workshops ( Figure 1 **A-F**). Workshop participants’ feedback clearly demonstrated a general resonance for the stakeholders with the previously published source material as well as shared experiences in the newly produced text. Some of the material was more specific, for example there was text that mentioned parental caring responsibilities, and so some participants connected more or less with this, but overall, it was strongly relatable for the groups that we shared it with. Throughout the workshop process, it became apparent that people reading, recounting and discussing the quotes was a meaningful process in itself. The embodied experience of reading out somebody else’s words had a simple, powerful resonance.

**Figure 1.**
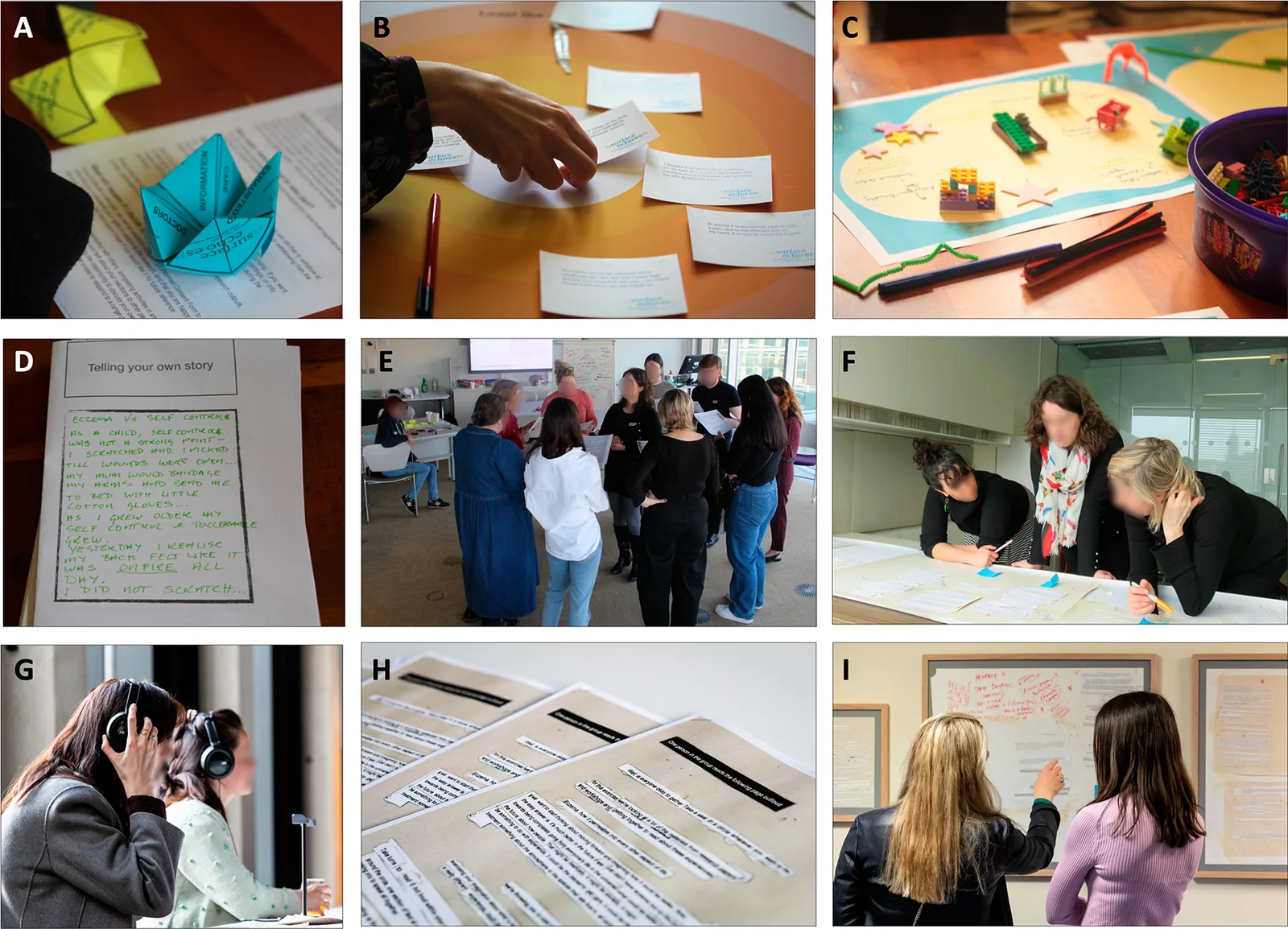
Participatory workshop activities, development of quotation collage and final works. Images blurred to de-identify individuals; **A** Chatterboxes; **B** Proximity circles and quotation cards; **C** Imaginary future island; **D** Storytelling; **E** Script reading; **F** Co-creation of text and quotation collage to form a script; **G**, **H,I** Final outputs: audio, script and framed collage (photos G, H and I by Sally Judd).

### Selection of quotations for use in the final script

Quotations selected for inclusion in the script encompass a wide range of lived experience, including misunderstandings with family, friends and healthcare professionals, profound challenges associated with sleep deprivation, questions and hopes for a better future. Specific themes presented in the work include hurtful comments about visual appearance and the mental burden of embarrassment and shame (“ *it’s a kind of social handicap during those periods when it is bad*”); pain as well as itch; lack of clarity around topical and other treatments (“ *I think this ‘trial and error’ is a very primitive form of medicine*”); the challenges of communicating effectively with healthcare professionals and the relapsing-remitting nature of the condition; and the morbidity associated with sleep deprivation (“ *It’s impossible to be on sick leave just because you are tired. So I carried on throughout the Spring and then I completely collapsed*”). The participants also reported positive and validating experiences: “ *I felt listened to … it was a conversation*” and their imagination of a better future, for example *“… consistent advice about caring for your skin*” and *“… taking the stress out of it*”.

### Script for group reading aloud and audio for group or individual listening

The audio version of the script ( Figure 1G) is available to listen to online, or to download as an mp3 file with a duration of 23 minutes. ^24^ The audio is intended for professionals and a wider audience who may not have the opportunity for group-reading, and for access purposes for those who may for any reason find reading to be a barrier. Colour ( Figure 1H) and grey-scale versions of the script are in PDF format for digital download or printing onto 20 sides of A4 paper. ^24^ The script is designed to be read together in a group, with a friend, or alone, but we have found it to be powerful when read out loud together in a group of 3–10 people, as a collective experience of reading and listening to other people’s voices.

### Online material

The art-science collaborative organisation ASCUS Art & Science hosts a blog in which the three years of work in creating “Surface Echoes” are recorded. ^25^ The script and audio resources are available free to download from the same website. ^24^

### Collage framed for public display

The original text collage has been framed as a set of 6 sections and is permanently displayed in the NHS Lothian Department of Dermatology (Edinburgh, Scotland, Figure 1I). Its placement within a corridor that is frequently used by patients, members of staff and healthcare professionals in training contributes to fulfilling a specific request by workshop participants for their voices to be amplified to these key stakeholders.

## Discussion

This work has been conducted to raise awareness, promote discussion, reduce stigma and increase understanding of eczema. The presentation to healthcare workers, research scientists and industrial funders was requested by the participants whose voices are amplified, to improve empathic discussion with professionals at all levels and inform future research. It was clear from early on in the project that people living with eczema in the UK experienced challenges in being listened to, and that they were seeking understanding and consistency in care. Much of the content within the original quotes that we drew from ^7– 22^ was centred on medicalised experiences, and we therefore steered towards a primary audience of healthcare professionals (doctors, nurses and pharmacists) working in dermatology and beyond, as well as medical and nursing students, and those working in community health. However, the material produced has been designed to maximise availability and accessibility to wide audiences.

The “Surface Echoes” script has been used in a variety of ways: as a training resource for healthcare professionals; by people wishing to learn more about what it’s like to live with eczema; and as a printable resource to share informally within families and between friends and colleagues, to stimulate discussion when it’s difficult to talk openly about eczema. The text is purposefully quite awkward to read; it maintains a vernacular tone, having come from verbatim transcription. It was important to our interdisciplinary team to keep the character of the natural spoken word. This form of script means that the words and phrases can’t be skimmed over in a fast-paced reading style that may be familiar to busy professionals. In our experience, it slows down the reading pace of even those who are familiar with the text. In this way, the script is purposefully designed to require the readers to pay attention. This is through both the vernacular character of the writing and also the turn-taking, meaning the readers have to stay focused on the text, so they don’t miss their turn. This turn-taking process is self-organised and negotiated by the group, in the round. The process of working through the script brings alive the collective spoken word and the embodied experience, aligning with the dancer and theorist Susan Kozel’s notion of resonance as a powerful phenomenological performative strategy. ^26^ The audio version can create a more intimate, immersive experience, particularly when listened to with headphones. The range of voices mirror the wide extent of people affected by eczema, which has impact across communities and different aspects of human life. We welcome anyone with a professional or personal interest in or experience of eczema to listen to the audio version of the script.

The output materials from “Surface Echoes” have been presented at academic conferences for artists, creative practitioners, qualitative researchers and healthcare audiences. It has been used by Eczema Outreach Support in community settings and is being integrated into undergraduate medical teaching at the University of Edinburgh. It has also been used by families to start conversations between parents and young people about their experience of eczema and healthcare, which demonstrates the project’s ability to create space for dialogue where words are often hard to find.

We report our experiences from this three-year patient and public engagement project to share points of learning. We also hope to inspire interdisciplinary groups to consider creative opportunities to amplify the voices of other under-represented groups throughout research and healthcare.

## Ethical considerations

The proposed work was reviewed and approved by the Edinburgh College of Art research ethics review board at the University of Edinburgh (response ID 193402–193395-102844277; 30 Jan 2023) and conducted in accordance with the principles of the Declaration of Helsinki.

## Consent

Individuals provided written informed consent before participation, and agreement that their work and words could be used for the development of a creative script for dissemination and publication. Consent has been obtained to publish the images in this manuscript and to share the source data.

## Data Availability

The “Surface Echoes” resources (PDF of participatory script and mp3 audio file) are available for download from the webpage https://www.ascus.org.uk/projects/surface-echoes . Anonymised source data are available via Edinburgh DataShare https://doi.org/10.7488/ds/8102 ^27^ under Creative Commons License: Attribution 4.0 International. Other original creative work was produced under contract for this project and is protected by copyright to the artists.
