# Supplementary material for: Creative patient and public engagement to amplify the voices of people living with eczema
Source: Wellcome Open Res. 2026 Sep 26;11:753. [Version 1] doi: 10.12688/wellcomeopenres.26101.1 (PMC13687730; doi:10.12688/wellcomeopenres.26101.1)
Supplement: Peer review history

# Reviewer report: Reviewer response for version 1

**DOI:** 10.21956/wellcomeopenres.28743.r170970

**Published:** 2026-10-21

**Authors:** Fiona Johnstone

**Affiliations:** 1 Durham University, Durham, UK

This research note clearly and cogently sets out the methods and findings of *Surface Echoes*, a creative and interdisciplinary public and patient engagement project exploring experiences of living with eczema. I found the article particularly useful at the level of method and process: the different creative approaches are clearly explained, and I can imagine this being genuinely useful to others seeking to develop similar participatory projects. The visual and audio outputs from the project, available on the ASCUS website, are also really engaging.

 I was particularly intrigued by what the authors call “material exploration”, and would have liked a little more detail about what this brought to the project in epistemological terms. For example, what kinds of objects or materials did participants choose to represent their experiences of eczema? How did the discussions elicited through materials differ from, extend or complicate the experiences already captured in the existing qualitative research? Some examples here would help to articulate more clearly what material methods bring to the table that more conventional qualitative approaches might not.

 I was also curious about the relatively small scale of the workshops, and would have liked to see some reflection on the benefits of working with groups of this size. What becomes possible through working with a relatively small group? Equally, what might be gained or lost by scaling this approach up to work with greater numbers of participants? This reflection would be particularly useful for readers who might want to adapt the methodology for their own projects.

 Finally, as an art and visual culture scholar, I appreciated the attention to aesthetic detail, particularly the decision to retain some of the awkwardness of verbatim speech in the resulting text. This means that the text cannot easily be skim-read: the reader has to slow down, pay attention and work hard to discern its meaning. This seems to me to be an important part of what the work is doing, rather than simply an aesthetic choice. I would therefore have liked to see this theorised a little more rigorously, perhaps by extending the discussion of resonance as a phenomenological and performative strategy.

 Overall, I found this a thoughtful, engaging and genuinely useful research note. The authors describe the project and its creative processes very clearly and, in their stated aim of sharing learning from the project, I think they succeed.

Is the work clearly and accurately presented and does it cite the current literature?

Yes

If applicable, is the statistical analysis and its interpretation appropriate?

Not applicable

Are all the source data underlying the results available to ensure full reproducibility?

Yes

Is the study design appropriate and is the work technically sound?

Yes

Are the conclusions drawn adequately supported by the results?

Yes

Are sufficient details of methods and analysis provided to allow replication by others?

Yes

Reviewer Expertise:

Art history and visual culture

I confirm that I have read this submission and believe that I have an appropriate level of expertise to confirm that it is of an acceptable scientific standard.

# Reviewer report: Reviewer response for version 1

**DOI:** 10.21956/wellcomeopenres.28743.r170972

**Published:** 2026-10-09

**Authors:** Allison R Loiselle

**Affiliations:** 1 National Eczema Association, Novato, California, USA

“Surface Echoes” was a three-year, patient and public engagement project in the UK that set out to move the lived experience of atopic eczema out of academic papers and in front of the people who most need to hear it. A multidisciplinary team of artists, researchers, and engagement specialists combined quotes from published qualitative studies with new material from participatory workshops, then collaged the text into a script. The outputs are a pdf, a 23-minute audio recording voiced by five actors, and a framed collage on permanent display in an Edinburgh dermatology department. The materials are free to download and have been used in healthcare professional training, medical teaching, community settings, and family conversations, with the authors offering the approach as a model for amplifying other under-represented voices.

 For those of us who work in the eczema space, it is a mountain of a task to capture themes and quotes that represent the experience of eczema, when each patient and caregiver has such a unique and personal experience. The tendency can be to go focus too narrowly or too broadly. The authors delicately navigated this problem and have created outputs that will be confirming to most patients, relevant to healthcare stakeholders, and enlightening for people who don't know anything about eczema or its impact.

 While I appreciate the creative and qualitative nature of this work, it would be interesting to quantify the use/downloads a bit more to demonstrate concrete impact. Questions like: How many people have downloaded the resources from the website? What stakeholder group are they in? Are they from outside the UK? Are there any testimonials from those who have used it for training/how it benefitted them? If this information is available and added to the discussion, I believe it would amplify the described impact.

Is the work clearly and accurately presented and does it cite the current literature?

Yes

If applicable, is the statistical analysis and its interpretation appropriate?

Not applicable

Are all the source data underlying the results available to ensure full reproducibility?

Yes

Is the study design appropriate and is the work technically sound?

Yes

Are the conclusions drawn adequately supported by the results?

Yes

Are sufficient details of methods and analysis provided to allow replication by others?

Yes

Reviewer Expertise:

eczema

I confirm that I have read this submission and believe that I have an appropriate level of expertise to confirm that it is of an acceptable scientific standard.
